# New Inventions

**Published:** 1895-11

**Authors:** 


### NEW INVENTIONS.

An appliance for milking cows, consisting of a series of tubes, provided at one end with teat-pieces, converging to a spout, leading to a pan or vessel, the whole suspended by a strap over the back.

A hitching-device, to be secured to the bottom of a vehicle and to be made secure, as needed by the driver on entering or leaving the vehicle, through a lever fastened to a swinging-step.

A soft-tread horseshoe having flanges along its inner and outer edges, said flanges being reduced at certain points, both the flanges and the reduced portions being serrated.

Case for surgical instruments, consisting of a tray covered with corrugated material and forming pockets.

A fly-catching device for animals, consisting of a framework to be fastened to the animal to which fly-catching compounds may be attached.

Veterinary obstetrical-forceps, consisting of a pair of oppositely-disposed jaws, having handled shanks inseparably pivoted together by a stud on one shank to slide loosely in a longitudinal slot in the other shank, whereby, one jaw may be advanced and inserted before the other.

An improved horse-stall, with homogeneous, non-absordent floor, with movable slats secured to hooks fastened in flooring; the other end fastened by a key-strip, the openings being free and allowing brooming out.

A hypodermic bulb-syringe, the bulb fastened to the barrel at one end and worked through a spring-controlled valve.

A foot-hook for cleaning horses' feet, consisting of a hoe-shaped part for cleaning dirt from the outside and bottom of foot, a hook for cleaning the frog, a hoof-knife for veterinary use, and a screwdriver-edge for farrier's and general use.

A bit, consisting of a mouthpiece, two rings at the ends of mouthpiece, a lever connected with each ring to swing relatively to the mouthpiece, with means for attachment at free ends of said levers to overdraw and in the middle portion to the chin-straps.

A safety-hook for stables, consisting of a body portion with a bolt therein carrying a swinging hook with an arm (the former parts rotating on each other), a catch on the body, open at one side, and a spring acting on the bolt.

A horse-blanket, or cover, with an open hood in two parts, to cover base of tail, to prevent the blanket working off the rump.

A combination pad and horseshoe, having the rear or tread portion thickened, the intermediate shouldered portion, the flat upper thin portion and the overlapping forward ends of the tread portion providing recesses for the ends of the shoe.

A combination hitching-post and chain, the latter running through the centre of the hollow post, on an automatic roller.

A creeper-shoe for horses, consisting of a combination plate of sharp, gritty material and rubber, a series of pivot-lugs embedded therein and projecting laterally from the edges of the plate ; forward and side strap-holders pivoted to the lugs, there connected with other straps destined to maintain the whole to the hoof.

A prescription-file, consisting of two uprights fastened to a suitable base, with file wires running between the uprights, at one upright rigid hooks and the other movable, adjustable wires with loops at the end to fasten to the hooks.

